# Characterization of Involuntary Contractions after Spinal Cord Injury Reveals Associations between Physiological and Self-Reported Measures of Spasticity

**DOI:** 10.3389/fnint.2017.00002

**Published:** 2017-02-09

**Authors:** Meagan Mayo, Bradley A. DeForest, Mabelin Castellanos, Christine K. Thomas

**Affiliations:** ^1^The Miami Project to Cure Paralysis, University of MiamiMiami, FL, USA; ^2^Department of Neurological Surgery, University of MiamiMiami, FL, USA; ^3^Department of Physiology and Biophysics, University of MiamiMiami, FL, USA

**Keywords:** muscle spasms, tone, wheelchair transfer, H-reflex, F-wave, muscle co-activation

## Abstract

Correlations between physiological, clinical and self-reported assessments of spasticity are often weak. Our aims were to quantify functional, self-reported and physiological indices of spasticity in individuals with thoracic spinal cord injury (SCI; 3 women, 9 men; 19–52 years), and to compare the strength and direction of associations between these measures. The functional measure we introduced involved recording involuntary electromyographic activity during a transfer from wheelchair to bed which is a daily task necessary for function. High soleus (SL) and tibialis anterior (TA) F-wave/M-wave area ratios were the only physiological measures that distinguished injured participants from the uninjured (6 women, 13 men, 19–67 years). Hyporeflexia (decreased SL H/M ratio) was unexpectedly present in older participants after injury. During transfers, the duration and intensity of involuntary electromyographic activity varied across muscles and participants, but coactivity was common. Wide inter-participant variability was seen for self-reported spasm frequency, severity, pain and interference with function, as well as tone (resistance to imposed joint movement). Our recordings of involuntary electromyographic activity during transfers provided evidence of significant associations between physiological and self-reported measures of spasticity. Reduced low frequency H-reflex depression in SL and high F-wave/M-wave area ratios in TA, physiological indicators of reduced inhibition and greater motoneuron excitability, respectively, were associated with long duration SL and biceps femoris (BF) electromyographic activity during transfers. In turn, participants reported high spasm frequency when transfers involved short duration TA EMG, decreased co-activation between SL and TA, as well as between rectus femoris (RF) vs. BF. Thus, the duration of muscle activity and/or the time of agonist-antagonist muscle coactivity may be used by injured individuals to count spasms. Intense electromyographic activity and high tone related closely (possibly from joint stabilization), while intense electromyographic activity in one muscle of an agonist-antagonist pair (especially in TA vs. SL, and RF vs. BF) likely induced joint movement and was associated with severe spasms. These data support the idea that individuals with SCI describe their spasticity by both the duration and intensity of involuntary agonist-antagonist muscle coactivity during everyday tasks.

## Introduction

Spasticity is defined classically as a velocity-dependent increase in resistance to passive stretch (Lance, 1980), but clinically it presents as increased muscle tone, hyperreflexia and/or involuntary muscle contractions (spasms; Adams and Hicks, 2005). Most individuals with chronic spinal cord injury (SCI; cervical: 78%–93%; thoracic: 45%–82%) have symptoms of spasticity, but fewer individuals report their spasticity as problematic (cervical: 36%–52%; thoracic: 24%–45%; Maynard et al., 1990; Sköld et al., 1999; Thomas and Field-Fote, 2009). When individuals with SCI are asked about spasticity, they almost always describe which muscles contract involuntarily and how these spasms either help them (e.g., to stand) or interfere with activities of daily living, sleep, and/or rehabilitation (Little et al., 1989; Adams and Hicks, 2005).

Electromyographic activity (EMG) has been used to document the various types of involuntary muscle contractions that occur during daily tasks (Tepavac et al., 1992; Winslow et al., 2009; Thomas et al., 2014), or imposed movements (Benz et al., 2005), when tone is assessed using the Ashworth scale (Sköld et al., 1998; Sherwood et al., 2000; McKay et al., 2004), as muscles are stretched passively (Burke et al., 1971), or when people with SCI walk (e.g., Yang et al., 1991; Harkema, 2001; Kressler et al., 2014). If injured individuals are using these contractions to judge spasticity, quantification of this muscle activity may provide a novel and important approach to improve the poor correlations often found between physiological, clinical and self-reported measures of spasticity. For example, individuals with spasticity had either pronounced or absent reciprocal inhibition but there was no difference in the degree of their spasticity when it was assessed clinically (tone, tendon reflexes, clonus; Crone et al., 1994). Self-reported spasm frequency scores correlated poorly with clinical assessment of perceived resistance against passive movement of the limb (i.e., tone using the modified Ashworth scale) or tendon reflexes (Priebe et al., 1996; Baunsgaard et al., 2016).

In this study, our first aim was to quantify three distinct measures of spasticity: functional (defined as the involuntary EMG generated during performance of a daily task that is a necessary function), self-report (spasm characteristics, tone), and classical physiological measures (H/M ratio (Hoffman reflex/maximal muscle compound action potential), low frequency H-reflex depression, F-wave persistence (late responses produced by antidromic activation of motoneurons in response to supramaximal nerve stimulation; Magladery and McDougal, 1950) and F-wave/M-wave (F/M) area ratio). Characterization of the involuntary EMG generated during the performance of an everyday task is new and was intended to capture features that individuals with SCI may use to describe their spasticity. A transfer from a wheelchair to a bed was chosen to represent a typical daily task because most people with SCI travel by wheelchair and complete transfers multiple times per day (Finley et al., 2005), movement is a frequent trigger for muscle spasms (Kawamura et al., 1989), and many individuals report that spasms interfere with transfers (Little et al., 1989). Our second aim was to examine the strength and the direction of the associations between the functional, self-reported and physiological assessments of spasticity after chronic SCI. If alterations in spinal inhibition (low frequency H-reflex depression) and/or motoneuron excitability (F/M area ratio, persistence) with SCI contribute to the generation, maintenance and magnitude of spasms in multiple muscles, and individuals with SCI are interpreting their spasticity in terms of the muscle spasms elicited during daily activities, associations would be expected between physiological, functional and self-reported measures of spasticity. These comparisons may improve our understanding of the complexity and scope of spasticity, how physiological processes drive muscle spasms, the attributes that individuals use to describe their spasticity, as well as which features have an impact on injured individuals and may need management.

## Materials and Methods

### Participants

Two groups of people were examined. Individuals with chronic (>1 year) thoracic SCI completed functional (*n* = 7), physiological (*n* = 12), and self-report (*n* = 12) measures of spasticity (Table 1). Nineteen uninjured controls (Un) completed only the physiological measures (6 women, 13 men, 19–67 years). Uninjured tibialis anterior (TA) and vastus lateralis (VL) maximal M-wave data were taken from an earlier study (13 men, 11 women; age: 19–51 years; Klein et al., 2010). Table 1 also summarizes the background information for the SCI population in order of increasing maximal H/M ratio. One participant with SCI was taking baclofen to control spasticity at the time of examination. The leg chosen for evaluation was the side reported to be most prone to spasticity (SCI, left: *n* = 6; right: *n* = 6). All experimental procedures were approved by the University of Miami Institutional Review Board (UM IRB) and all participants (SCI and Uninjured) gave informed, written consent prior to participation even though the uninjured data came from an earlier study which involved a different IRB protocol.

**Table 1:** **Spinal cord injury (SCI) participant demographics**.

Participant	Sex	Age(years)	SCI duration (years)	SCI level	SCI cause	AIS	Lumbar-sacral light touch/pin prick score	^Δ^Clonus/Other spasms
1*	M	25	7	T11	Gunshot	A	0/0	No/No
2	F	47	28	T7	MVA	A	0/0	Yes/Yes
3^†^	M	52	27	T8	MVA	B	11/9	Yes/Yes
4	F	37	2	T3	MVA	A	0/0	Yes/Yes
5^†*^	M	26	1	T8	Fall	A	0/0	No/Yes
6^†^	M	36	21	T5	Stab wound	B	4/4	Yes/Yes
7^†^	M	28	1	T2	MVA	B	4/4	Yes/Yes
8	M	45	13	T10	Gunshot	A	0/0	Yes/Yes
9^†#^	F	23	2	T10	MVA	B	4/4	Yes/Yes
10^†^	M	19	2	T2	MVA	A	0/0	Yes/Yes
11^†^	M	20	5	T5	MVA	A	0/0	Yes/Yes
12	M	26	1	T7	Gunshot	A	0/0	Yes/Yes

*No self-reported spasms; ^†^Transfer recorded; M, male; F, female; T, thoracic; MVA, motor vehicle accident; SCI level and completeness were assessed according to the International Standards for Neurological and Functional Classification of Spinal Cord Injury and the American Spinal Injury Association Impairment Scale (AIS); ^Δ^Self-triggered clonus and other spasms; ^#^80 mg/d baclofen.

### EMG Recordings

**Figure 1:**
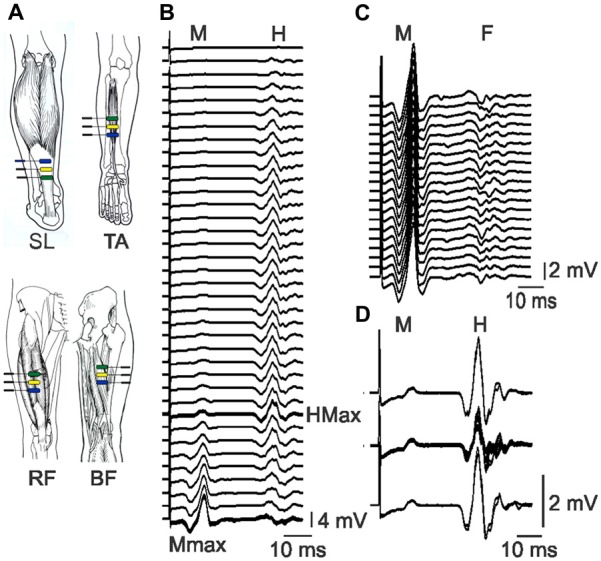
**Electrode placement and physiological data. (A)** Bipolar EMG was recorded from soleus (SL), tibialis anterior (TA), rectus femoris (RF) and biceps femoris (BF) with electrodes ~4 cm apart. Pictures of muscles used with permission (Sieg and Adams, 2009). **(B)** Raster of SL EMG evoked by tibial nerve stimulation at increasing intensity (top to bottom). The H-reflex increases, then decreases as the M-wave increases. The maximal H-reflex trace is thick. Maximal M-waves are overlaid. **(C)** Raster of maximal SL M-waves and the associated F-waves which vary across responses. **(D)** Overlay of control SL M-waves and H-reflexes (top and bottom, two traces each) and M-wave and H-reflexes evoked in response to pulses at 1 Hz stimulation (middle). The M-waves (2.5 ± 0.6% Mmax) remained stable throughout. All data are taken from participant 6.

Surface EMG was recorded from soleus (SL), TA, the biceps femoris (BF) head of hamstrings, and the rectus femoris (RF; for SCI) or VL (for Uninjured) head of the quadriceps using adhesive electrodes (First Choice 2000 electrodes, trimmed to ~1 cm, Tyco Healthcare, Princeton, NJ, USA). Three electrodes were placed on each muscle, ~4 cm apart (Figure 1A). The two distal electrodes were active and the proximal electrode was a ground for all muscles (Klein et al., 2010), except SL, where the distal electrode was the ground. For RF and VL, the distal electrode was placed ~12 cm proximal to the patella. The distal electrode for BF was aligned with the middle RF or VL electrode but on the midline of the posterior surface of the leg. The distal TA electrode was placed on the muscle ~1 cm proximal to the tendon. The proximal SL electrode was approximately ~3 cm distal to the medial gastrocnemius muscle. The electrodes were secured to the skin with Hypafix tape (Smith and Nephew, Andover, MA, USA). EMG signals were amplified (P511 amplifier; Natus Neurology-Grass, West Warwick, RI, USA), filtered (30 Hz to 1 kHz), and sampled to a computer at 3125 Hz (1401 interface, Spike2, Cambridge Electronic Design, Cambridge, England).

### Experimental Protocol

#### Functional Measure of Spasticity

**Figure 2:**
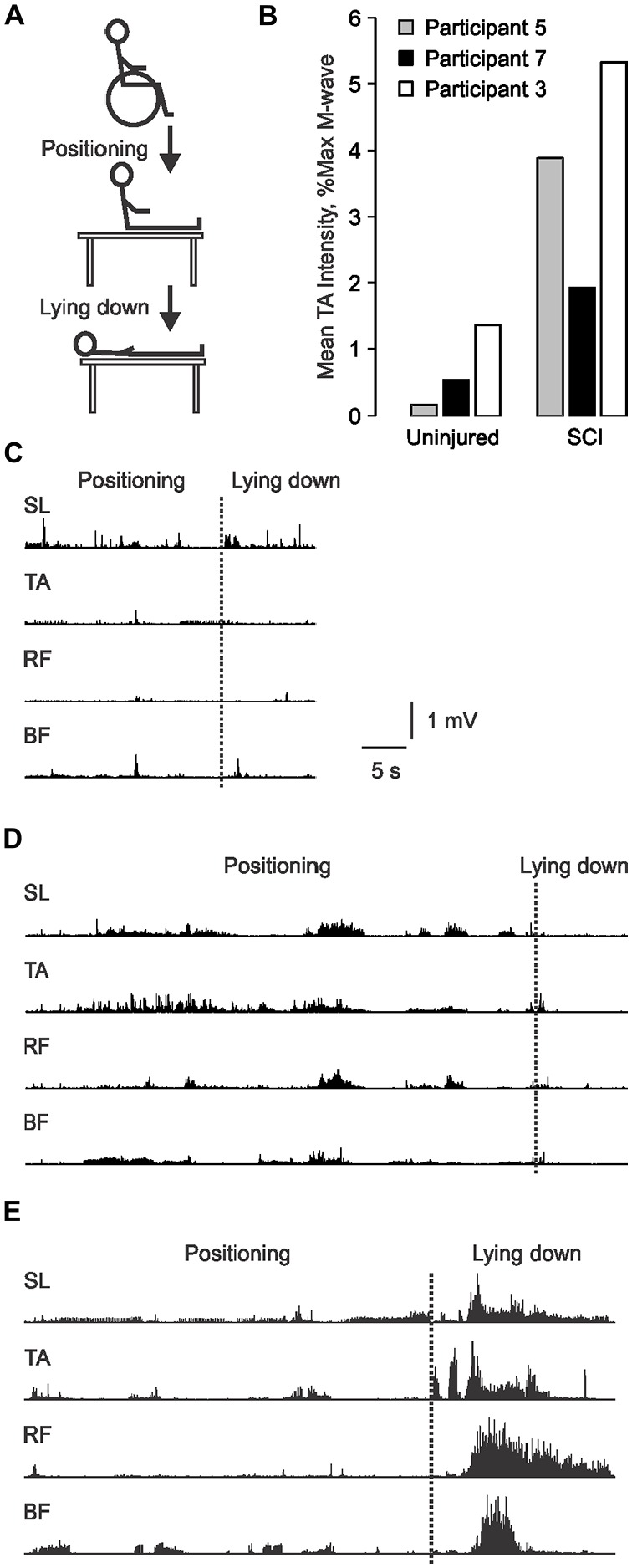
**Involuntary EMG generated during transfers from a wheelchair to a bed. (A)** Schematic of the transfer task. **(B)** TA EMG intensity for the data shown in **(C–E)** normalized to Uninjured and spinal cord injury (SCI) maximal M-wave data. EMG recorded from SL, TA, RF and BF as participant 5 **(C)**, 7 **(D)** and 3 **(E)** transferred from their wheelchair to our bed (positioning), then lay down. The transition between the two phases is marked by a dotted line.

The involuntary EMG produced in leg muscles during a daily task that is a necessary function was recorded and used as a functional measure of spasticity. This task required eight participants with SCI to transfer themselves from their wheelchair to the examination bed, then to lie down using their typical movement strategy (Figure 2A). One participant was unable to transfer without assistance, so data were collected from seven participants. The task was designed to mimic an individual’s natural movement. Therefore, displacement distances and speed of movement were not standardized across participants or experiments.

#### Physiological Measures of Spasticity

Changes in motoneuron excitability and reductions in various kinds of inhibition are important contributors to spasticity (D’Amico et al., 2014), and are assessed here using F-waves, H-reflex amplitude, and modulation. Participants were tested supine with the test leg bent at the knee (110°) and the ankle (150°). The foot was placed flat on the bed, but a slight incline of the bed’s foot rest kept the leg from sliding out of position. The knee was held in place by an experimenter to prevent leg movement during stimulation. The only exception was for recording muscle compound action potentials from RF or VL, when the leg was extended fully.

*Maximal muscle compound action potentials (M-waves)*, a measure of maximal muscle output, were recorded from SL, TA, and RF or VL muscles in response to supramaximal stimulation of the tibial, common peroneal, and femoral nerves, respectively (~20% higher than the intensity that evoked a maximal M-wave; DS7AH, Digitimer, Ltd., Welwyn Garden City, UK). The optimal site for stimulation of each nerve was determined by adjusting the placement of the stimulating electrode until the M-wave was maximized for a given stimulation intensity. Single pulses (pulse width: 200 μs pulses for femoral and tibial nerves; 50 μs pulses for common peroneal nerves) were delivered to each nerve, 8 s apart, starting below threshold (no evoked EMG) and increasing in intensity (1 or 5 mA steps) until a maximal M-wave (Mmax) was attained (no increase in M-wave with increments in stimulus intensity; Figure 1B). The sequence in which the muscles were studied was altered across experiments to minimize any order effects.

##### F-waves

To examine F-wave size and persistence, both measures of motoneuron excitability (Butler and Thomas, 2003), 20 pulses at 1 Hz were delivered to the tibial and common peroneal nerves at supramaximal intensity. EMG were recorded from SL and TA muscles, respectively (Figure 1C). F-waves were also analyzed from the subset of pulses that evoked maximal M-waves when stimulus intensity was increased progressively (mean ± SD number of pulses for SCI: 25 ± 8; Un: 28 ± 8). Since TA motoneurons are generally less excitable than SL motoneurons (Espiritu et al., 2003), TA F-waves were also examined to avoid the ceiling effect in SL F-wave persistence.

##### Soleus H-reflexes and low frequency H-reflex depression

To examine the excitability of the monosynaptic reflex pathway, H-reflexes were recorded from SL muscles during the same series of pulses delivered to evoke M-waves (Figure 1B). When the maximal H-reflex was expressed relative to the maximal M wave, the H/M ratio indicated the fraction of motoneurons that were excited reflexly by the nerve stimulation. We found similar H-reflex amplitudes and H/M ratios for two participants with pulse durations of 200, 500 or 1000 μs, so the shorter pulse duration was used due to less subject discomfort. To examine depression of the H-reflex in response to 1 Hz stimulation (post activation depression at Ia-motoneuron synapses, a measure of spinal inhibition; Schindler-Ivens and Shields, 2000), the intensity of the tibial nerve stimulation was adjusted until the evoked H-reflex amplitude was 50–60% maximal on the rising phase of the H-reflex curve (SCI: 64 ± 15% Hmax; Un: 59 ± 10%). A pair of pulses was then delivered 8 s apart (control pulses), followed by a train of 11 pulses at 1 Hz (test pulses), then another pair of control pulses (Figure 1D). The entire sequence was repeated if the evoked M-waves or the control H-reflexes were unstable in amplitude.

###### Maximum voluntary contractions (MVCs)

Each SCI participant was asked to produce three (5 s) maximal voluntary contractions of each muscle, 1 min apart. A tone from the computer was the cue to start and end the contraction. No voluntary EMG signals were recorded from individuals with SCI which signified that all of the leg muscles were paralyzed completely.

#### Self-Reported Spasticity

Individuals with SCI were asked to rate their spasticity throughout a typical day once in terms of: (1) spasm frequency (0: no spasms; 1: spasms induced only by stimulation; 2: infrequent spontaneous spasms occurring less than once per hour; 3: spontaneous spasms occurring more than once per hour; 4: spontaneous spasms occurring more than 10 times per hour); (2) spasm severity (0: mild; 1: moderate; 2: severe); (3) whether their spasms elicited discomfort or pain (0: none; 1: moderate; 2: severe); (4) spasm interference with function (0: none; 1: makes function difficult; 2: prevents function); and (5) muscle tone (0: no increase in tone; 1: slight increase in tone giving catch when the limb is moved in flexion or extension; 2: more marked increase in tone, but limb easily flexed; 3: considerable increase in tone, passive movement difficult; 4: limb rigid in flexion or extension) based on scales used by Priebe et al. (1996) with one exception. Participants rated their tone, instead of an experimenter performing the analysis. If clonus did not occur during the experiment, participants were also asked if they experienced this type of spasm, a typical clinical sign of spasticity.

### Data Analysis

All data were analyzed off-line using Spike 2 software (Cambridge Electronic Design).

#### Functional Measure of Spasticity

The EMG recorded from each muscle during a transfer from a wheelchair to a bed were rectified and integrated every 500 ms.

*Total transfer time* was from the instruction to start the transfer until the participant was supine on the bed and the EMG activity in all four leg muscles had either stopped or involved the firing of only one motor unit.

*EMG Duration* was the total time the EMG integral was above threshold during the transfer (mean + 3 SD of quiet baseline). EMG duration for each muscle was presented as a percentage of the total transfer time because the time it took to transfer varied across participants. *Co-activity* across the ankle and knee joint was assessed from the EMG measured from the SL and TA muscle pair, and the RF and BF muscles, respectively. *Coactivity by time* was calculated in two ways: (1) the percentage of transfer time during which a pair of muscles was activated together (above-threshold); and (2) the percentage of transfer time when one muscle of a pair had higher EMG than its antagonist.

*EMG intensity* was the mean amplitude of the EMG during the transfer. Above-threshold integrals for each muscle were time-normalized then expressed as a percentage of the average uninjured time-normalized maximal M-wave (Mmax) value for the respective muscle. Decrements in the maximal M-wave after SCI largely reflected muscle atrophy because M-wave amplitude was not correlated significantly with age and mean body mass index was similar for SCI (mean ± SD, range: 22.7 ± 3.2 kg/m^2^, 17.2–27.7 kg/m^2^) and Uninjured participants (23.6 ± 3.3 kg/m^2^, 17.4–28.6 kg/m^2^) suggesting comparable adipose tissue under the recording electrodes. Thus, when SCI data were normalized to uninjured means for men and women, it accounted for varying degrees of atrophy and sex-related differences in maximal M-wave values. Data from RF were normalized to uninjured VL values, as were BF data, since the sciatic nerve is too deep to stimulate reliably from the skin surface. This normalization is reasonable given that atrophy in hamstrings and different heads of the quadriceps muscles is similar after SCI (Castro et al., 1999). EMG were normalized to Mmax values rather than maximum voluntary contractions (MVC) data to avoid issues of submaximal voluntary activation in uninjured participants.

Initially, we normalized the EMG values during transfers to each participant’s maximal M-wave. However, this normalization approach shows EMG intensity in relation to the capacity of each muscle and fails to account for muscle atrophy. To illustrate this situation, we have compared the TA EMG generated during a transfer by three participants using the two normalization approaches. The absolute EMG generated in TA during the transfer was weak for participant 5 (Figure 2C), intermediate for participant 7 (Figure 2D), and greatest for participant 3 (Figure 2E). When normalized to Uninjured data, the respective EMG intensities maintained the same rank (0.17%, 0.52% and 1.36% maximal) because atrophy was accommodated (Figure 2B). However, when normalized to the maximal M-wave of each muscle, TA EMG intensity was 3.88%, 1.93%, and 5.33% maximal, respectively. This meant that the EMG intensity was 23 times larger for participant 5 because his maximal TA M-wave was of very low amplitude, but four times larger for the other two participants. Since our intent was to describe the relative intensity of contractions during a transfer in terms of an everyday task which must be performed by atrophied muscles after SCI, we normalized EMG intensity to uninjured data. This approach also allows direct comparisons between the participant groups.

*Co-activity for intensity* was calculated using ratios of the EMG intensities for the SL/TA and RF/BF muscle pairs for each 500 ms of the transfer:

$$\frac{\text{Extensor}\;\text{intensity}\;-\;\text{Flexor}\;\text{intensity}}{\text{Extensor}\;\text{intensity}\;+\;\text{Flexor}\;\text{intensity}}\times \text{Max }(\text{Extensor intensity or Flexor intensity})$$

The Max term is the greater of the two muscle intensities (SL or TA intensity for the ankle muscles; RF or BF intensity or the knee muscles), and was included because the peak intensities of opposing muscles did not always occur at the same time. Thus, this ratio gives an estimate of the intensity of the EMG in the two muscles relative to each other at a given time. From a functional point of view, movement may occur if one muscle is activated at a higher intensity than the other. When the ratio is positive, the extensor is activated more intensely; when the ratio is negative, the flexor EMG intensity is higher; when the ratio is zero, the two muscles have EMG of similar intensity. The greater the absolute value of the ratio, the greater the EMG in a given muscle relative to its antagonist. The *median ratio and peak (maximum) ratio for the extensor (ratio > 0) and flexor (ratio < 0) muscle of each pair* were determined for each transfer.

#### Physiological Measures of Spasticity

##### Maximal muscle compound action potentials (M-waves) and H-reflexes

The peak-to peak amplitudes of all M-waves and H-reflexes were measured. The maximal H-reflex (Hmax) was normalized to the maximal M-wave to provide the H/M ratio.

##### F-waves

The SL and TA EMG signals were filtered to separate the end of the maximal M-wave from the start of the F-waves (200–1000 Hz; Khan et al., 2012). Each F-wave was measured (area, time-normalized), then normalized to the respective M-wave area (time-normalized). The mean F/M area ratio was calculated for each muscle. F-wave persistence was the percentage of stimuli when an F-wave was present with a maximal M-wave.

##### Low frequency H-reflex depression

The peak-to peak amplitudes were measured for all control M-waves and H-reflexes, and those evoked by 1 Hz stimulation. The amplitude of the M-waves evoked by the control pulses and the 1 Hz pulses did not differ significantly (mean ± SD control vs. 1 Hz SCI: 9.8 ± 12.5% Mmax vs. 10.5 ± 12.6%; Un: 6.1 ± 6.0% Mmax vs. 7.0 ± 6.3%), indicating that similar numbers of motoneurons were recruited during the entire pulse sequence. Low frequency H-reflex depression was calculated as the mean amplitude of the H-reflexes evoked by 1 Hz stimulation, expressed as a percentage of the mean amplitude of the control H-reflexes. A value of 100% would indicate no low frequency H-reflex depression, while a value of 60%, for example, would indicate that the amplitude of the H-reflexes evoked by 1 Hz stimulation was depressed to 60% of the control H-reflex amplitude.

### Statistics

Mean and SD were calculated for each measure when a participant completed two experiments 1 month apart, otherwise a single value was used. Spearman rho correlations were used to examine associations between physiological, functional (transfer), self-report and demographic data. Group (SCI, Un) means and SDs were calculated for SL H/M ratio, low frequency H-reflex depression, F-wave area, and F-wave persistence. SCI data within two SDs of the Uninjured mean (95% of data) were designated as neutral, whereas data above or below this cutoff were considered high or low values, respectively. Chi square tests were used to compare the proportions in each designation across groups. Mann-Whitney U tests were used to compare group medians for these four variables, and TA F/M area ratio and F-wave persistence, because the data were not normally distributed. There were no significant differences in EMG durations (*p* = 0.357), intensities (*p* = 0.102), or co-activity (*p* = 0.189; 2-way Repeated Measures ANOVAs) during different phases of the transfer (i.e., transferring the body from the chair to the bed vs. lying down on the bed). Therefore, data are only presented for the entire transfer. Further, participants viewed the entire transfer as one task. All statistical tests were performed using SPSS v22 (IBM; Armonk, NY, USA), with statistical significance set at *p* < 0.05.

## Results

### Functional Measure of Spasticity

The involuntary EMG signals generated during a necessary daily task (transfer from a wheelchair to a bed) were recorded once at the start of one (*n* = 3) or two experiments (*n* = 4) in those individuals with SCI who were capable of unassisted transfers as a functional measure of spasticity. Total transfer time ranged from 33.0 to 73.5 s (61.8 ± 17.2 s, *n* = 7). The leg muscles were quiet prior to the transfer (no EMG) so the EMG signals recorded from the SL, TA, RF and BF muscles of each participant during each transfer were initiated by the transfer. Triggers may have included passive joint movement, touch of the skin, and/or contact with surrounding objects. Both the duration and the intensity of the EMG during transfers varied across muscles and participants (Figures 2C–E). Most of the EMG was tonic or involved trains of motor unit potentials. Clonus (<10 beats) was seen in two participants. Co-activation of muscles was common.

#### EMG Duration

**Figure 3:**
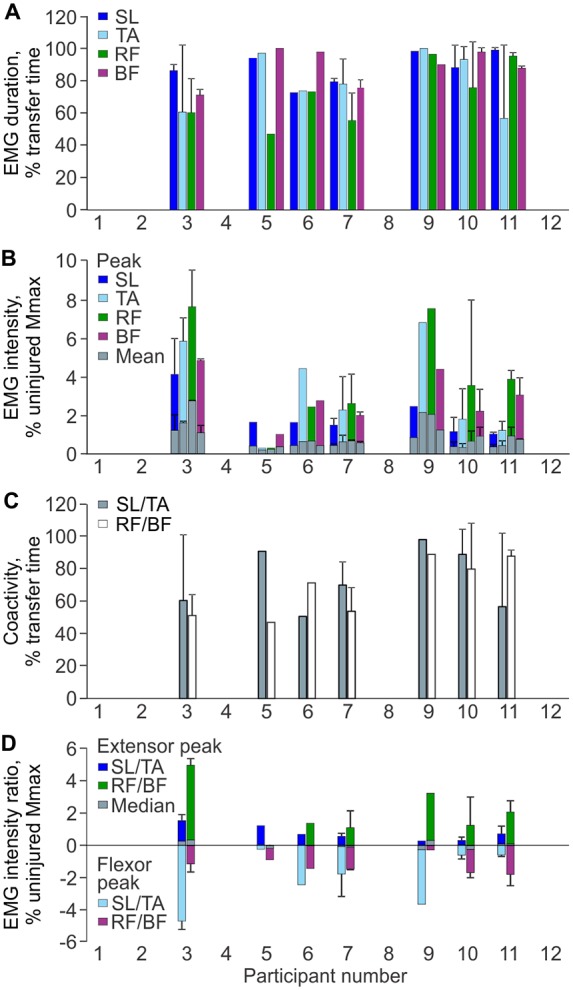
**EMG parameters during transfers. (A)** Mean (+SD) EMG duration for each muscle and participant. **(B)** Mean and peak (+SD) EMG intensity as a percentage of the average uninjured maximal M-wave by gender. **(C)** Mean (+SD) percentage of transfer time SL was coactive with TA, and RF was activated with BF by participant. **(D)** Median and peak intensity EMG ratios for SL/TA and RF/BF muscle pairs (positive values indicate the extensor values for the muscle pair; negative values indicate the flexor values; zero indicates EMG of equal intensity in both the extensor and flexor). Mean ± SD Uninjured vs. SCI maximal M-waves: SL Men: 23.4 ± 6.8 mV vs. 10.8 ± 4.8 mV, Women: 22.6 ± 7.2 mV vs. 5.8 ± 1.6 mV; TA Men: 16.2 ± 3.2 mV vs. 6.2 ± 4.8 mV, Women: 11.8 ± 2.6 mV vs. 4.4 ± 1.0 mV; VL (Un) or RF (SCI) Men: 13.6 ± 5.2 mV vs. 9.8 ± 7.6 mV, Women: 7.2 ± 2.0 mV vs. 5.4 ± 0.6 mV.

Each muscle was active during most of the transfer. Between participants, SL was active from 73% to 99% of the total transfer time, TA from 57% to 100%, RF from 47% to 96%, and BF from 71% to 100% (Figure 3A). Mean EMG duration did not differ across muscles (*p* = 0.205).

#### EMG Intensity

Average SL intensity ranged from 0.3% to 1.0% uninjured Mmax (peak: 1.0%–4.1%), TA from 0.2% to 1.8% (peak: 0.3%–6.8%), RF from 0.2% to 2.3% (peak: 0.3%–7.6%), and BF from 0.3% to 1.1% (peak: 1.0%–4.8%; Figure 3B). Neither mean nor peak EMG intensities differed between muscles (*p* = 0.108 and 0.094, respectively).

#### Co-Activity by Time

Co-activity between extensor and flexor muscles was prevalent throughout the transfer. Between participants, co-activity between SL and TA occurred from 51% to 98% of the transfer time, and for RF and BF from 47% to 89% of time (Figure 3C). The median time over which one muscle was activated more intensely than its antagonist tended to be greater for SL than TA (69% vs. 31% of transfer time, respectively), whereas this time was similar for RF and BF (51% vs. 49% of transfer time).

#### Intensity of Co-Activity

The median intensity for individual extensor/flexor EMG ratios remained relatively close to zero during the transfer, indicating that both muscles were activated at similar intensity (Figure 3D). For SL/TA, the group median for the flexor peak (TA) was −1.7% uninjured Mmax (range: −0.2% to −4.7%) while the extensor peak (SL) was only 0.6% (range: 0.3% to 1.3%). For the RF/BF ratio, the group median for the extensor peak (RF) was 1.4% uninjured Mmax (range 0.01% to 4.7%) while the flexor peak (BF) was −1.4% (range: −0.3% to −1.8%).

#### Correlations within Transfer

Intense EMG in RF was associated with high intensity EMG in BF (ρ = 0.786, *p* = 0.036) and TA (ρ = 0.893, *p* = 0.007). Other associations did not reach significance.

### Physiological Measures of Spasticity

Physiological measures were examined once in one (SCI: *n* = 4 participants, Un: *n* = 13) or two experiments (SCI: *n* = 8, Un: *n* = 6) and included: (i) SL H/M ratio which measures the reflex excitability of motoneurons in response to peripheral nerve stimulation; (ii) SL low frequency H-reflex depression, a reflection of post-activation depression (inhibition) at the 1a-motoneuron synapse; and (iii) SL and TA F/M area ratio and persistence, both measures of motoneuron excitability.

#### H/M Ratio

**Figure 4:**
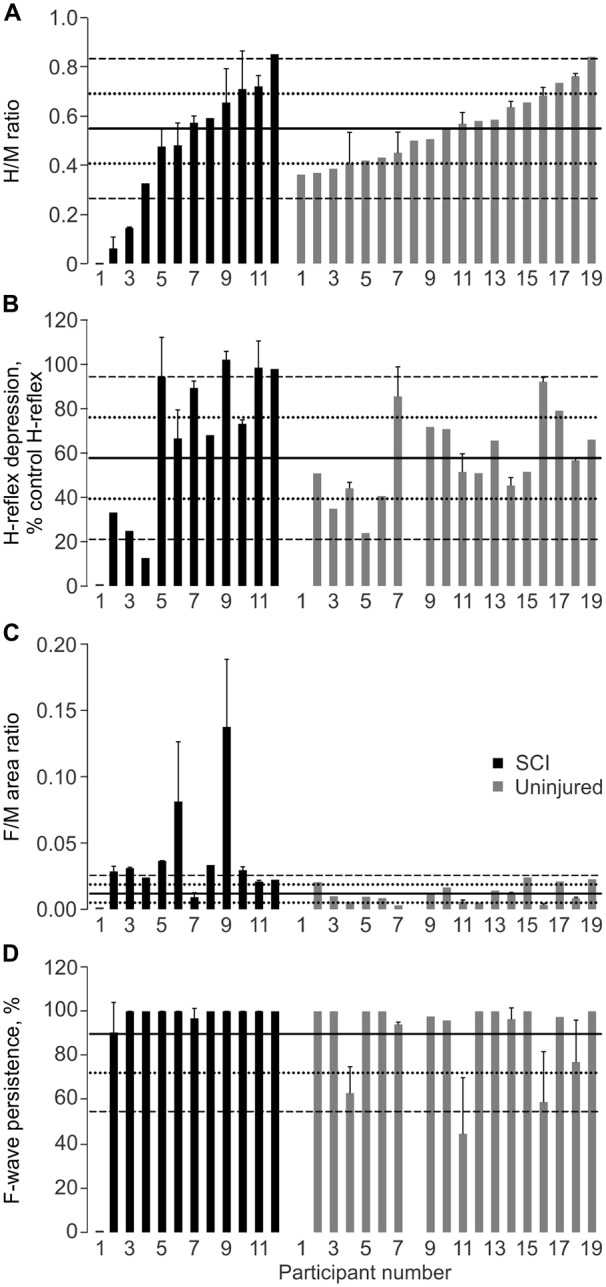
**Physiological measures of spasticity.** Mean + SD SL H/M ratio **(A)**, H-reflex depression at 1 Hz **(B)**, F/M area ratio **(C)**, and F-wave persistence **(D)** for SCI and Uninjured participants. Data for each group are plotted from low to high H/M ratio in this figure and other figures. No M-wave was evoked in one participant with SCI (participant 1). Solid, dotted and dashed lines show the Uninjured mean, ±1 SD, and ±2 SD, respectively. Mean ± SD Uninjured H/M Ratio: 0.55 ± 0.14; H-Depression: 57.8 ± 18.4%; F/M Area Ratio: 0.01 ± 0.01; F-wave Persistence: 89.7 ± 17.6%.

The median H/M ratios did not differ across groups (*p* = 0.425). When the data were ranked from low to high H/M ratio, hyperreflexia (H/M ratio greater than two standard deviations above the uninjured mean) was present in 1 of 11 SCI participants (Figure 4A). Hyporeflexia (<uninjured mean − 2SD) was present in two SCI participants. The remaining eight participants fell within two SDs of the uninjured mean. The proportion of data in each designation did not differ by group. The M-wave amplitude was comparable for each group when the H-reflex was maximal (mean ± SD SCI: 19 ± 21% Mmax; Un: 16 ± 12%; *p* = 0.899 Mann-Whitney), as found earlier (Ashby et al., 1974), so varying amounts of collision between antidromic impulses in motor axons and reflex responses does not explain the hyporeflexia. In another participant with thoracic SCI, no EMG responses were elicited in SL, even with long duration pulses, so the muscle was probably denervated completely.

#### Low Frequency H-Reflex Depression

The median values for low frequency H-reflex depression did not differ across groups (*p* = 0.161). Reduced low frequency depression was present in 3 of 11 SCI participants (Figure 4B). One person with SCI displayed greater low frequency H-reflex depression than in the Uninjured (mean − 2 SD). There were no significant differences in the proportion of data in each designation between groups (*p* = 0.106).

#### F-Waves

The median SL F/M area ratio was greater for the SCI group than the Uninjured (*p* = 0.001). Seven of 11 SCI participants had SL F/M area ratios greater than two SDs above the uninjured mean, but no participants had values more than two SDs below the mean (Figure 4C). The proportion of data in each designation differed for the SCI and Uninjured groups (*p* < 0.001).

The median values for SL F-wave persistence did not differ across groups (*p* = 0.388), largely due to a ceiling effect. All SCI participants had SL F-wave persistence within two SDs of the uninjured mean (Figure 4D). The proportions in each designation were not significantly different between groups (*p* = 0.580).

The median TA F/M area ratio was significantly higher after SCI (SCI: 0.033; Un: 0.010; *p* = 0.029), but persistence did not differ (median SCI: 58.2%; Un: 95.8%; *p* = 0.185).

#### Correlations within Physiological Measures

**Figure 5:**
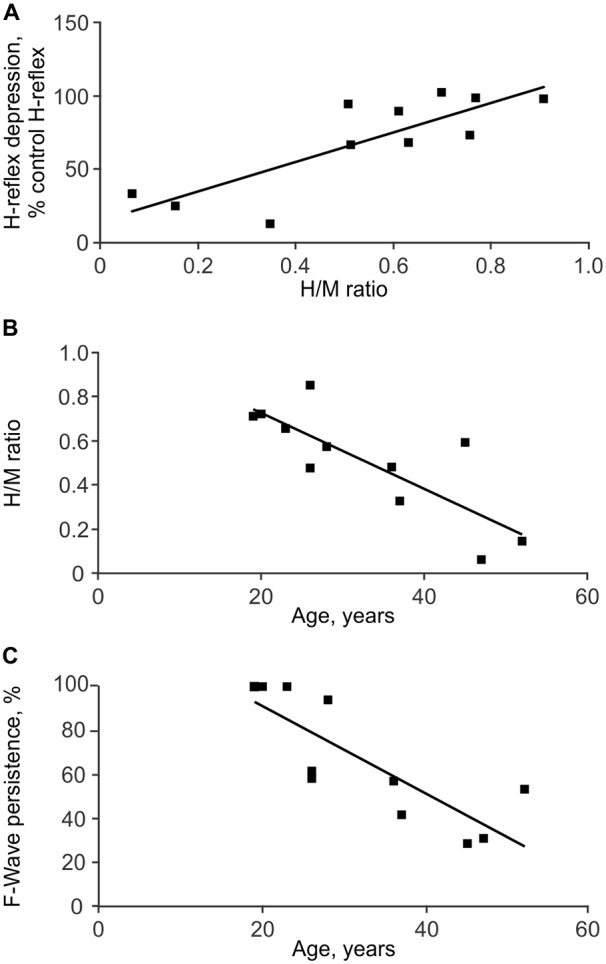
**Associations between physiological measures of spasticity.** Depression of the SL H-reflex in response to 1 Hz stimulation was less with greater H/M ratios **(A)**. SL H/M ratio **(B)** and TA F-wave persistence **(C)** both decreased with age.

High H/M ratios were associated with reduced low frequency H-reflex depression for each group (SCI: ρ = 0.76, *p* = 0.006, Figure 5A; Un: ρ = 0.56, *p* = 0.019). High H/M ratios and less low frequency H-reflex depression among SCI individuals were also associated with high TA F-wave persistence (ρ = 0.70, *p* = 0.022 and ρ = 0.76, *p* = 0.006, respectively) suggesting the general level of excitability was correlated across muscles. Associations between the other physiological measures of spasticity were not significant. However, in SCI participants, older age was associated with low H/M ratios (ρ = −0.78, *p* = 0.005; Figure 5B), more low frequency H-reflex depression (ρ = −0.78, *p* = 0.005), and low TA F-wave persistence (ρ = −0.897, *p* < 0.001; Figure 5C). Age did not significantly correlate with the F/M area ratios or SL F-wave persistence.

### Self-Reported Measures of Spasticity

**Figure 6:**
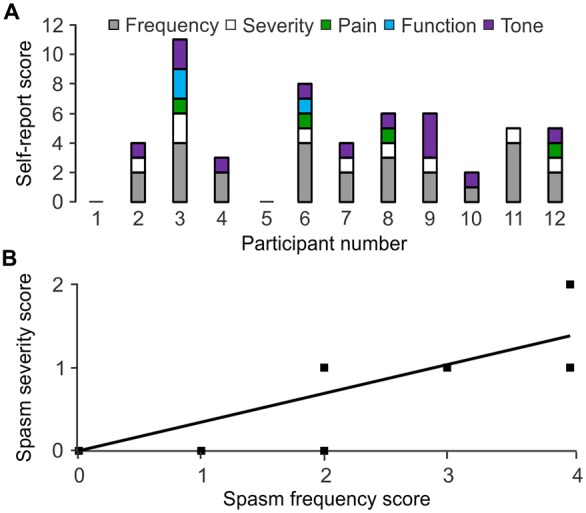
**Self-reported spasm and tone scores. (A)** Scores assigned to spasm frequency, severity, pain, interference with function, tone by participant. Higher numbers represent greater impairment. Participant 1 and 5 reported no spasms or tone. **(B)** Spasm frequency association with severity.

Participants rated their spasm frequency, severity, pain, interference with function and tone. Frequency of spasms was given the maximal rating (more than 10 times per hour) by 4 of 12 participants. One participant reported spasms occurring between one and nine times per hour, five said their spasms occurred infrequently (less than once per hour), while the remaining two reported never experiencing spasms including clonus (Figure 6A; Table 1). Only one participant considered their spasms severe, seven rated them as moderate, and four scored them as mild. Four participants indicated their spasms caused “mild discomfort or pain”, while eight experienced no discomfort or pain. One participant reported considerable increase in tone, making passive movement difficult, while eight reported an increase in tone that did not markedly effect passive movements, and three reported no increase in tone at all. Finally, one participant reported that spasms prevented function, one said his spasms made function difficult, and the remaining 10 participants indicated that spasms did not interfere with function.

#### Correlations within Self-Report

High spasm frequency was associated with strong spasm severity (ρ = 0.745, *p* = 0.008; Figure 6B). High spasm frequency, pain, and the combined scores for frequency and pain were associated with greater interference with function (ρ = 0.629, *p* = 0.038; ρ = 0.620, *p* = 0.042; ρ = 0.679, *p* = 0.022, respectively).

### Correlations between Measures

If the physiological, functional, and self-reported measures of spasticity assess overlapping features of spasticity, significant associations would be expected between these measures.

#### Transfer and Physiology

**Figure 7:**
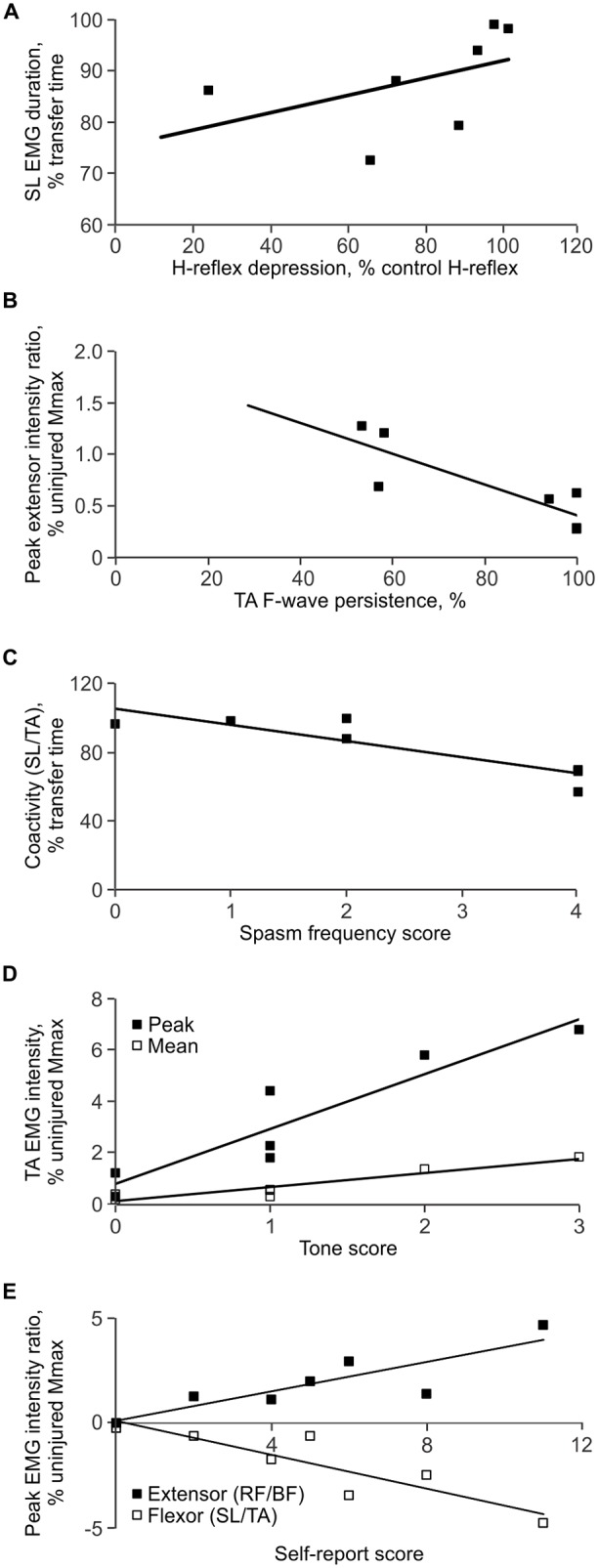
**Correlations between measures. (A)** Less low frequency H-reflex depression was associated with long SL EMG duration during the transfer. **(B)** Low TA F-wave persistence was associated with intense EMG in SL vs. TA, expressed as a higher peak extensor EMG ratio (SL EMG intensity < TA EMG intensity). **(C)** High spasm frequency was associated with decreased coactivity between SL and TA. **(D)** Greater self-reported tone was associated with strong mean and peak EMG in TA. **(E)** Greater total self-reported scores were associated with strong EMG in TA vs. SL, and strong EMG in RF vs. BF.

Decreased SL low frequency H-reflex depression was associated with long duration SL EMG during the transfer (ρ = 0.786, *p* = 0.036; Figure 7A). Increased TA F/M area ratio was associated with long duration BF EMG (ρ = 0.757, *p* = 0.049). Low TA F-wave persistence occurred when the intensity of EMG was stronger in SL than TA (a high extensor peak EMG ratio for SL/TA; ρ = −0.852, *p* = 0.015; Figure 7B).

#### Transfer and Self-Report

##### Frequency

Short duration TA activity during transfers (ρ = −0.805, *p* = 0.029), decreased coactivity (time) between SL and TA (ρ = −0.767, *p* = 0.044; Figure 7C), as well as between RF and BF (ρ = −0.755, *p* = 0.05), were all associated with high ratings for spasm frequency.

##### Severity

Intense EMG in TA (mean: ρ = 0.797, *p* = 0.032), RF (mean: ρ = 0.777, *p* = 0.040), and BF (peak: ρ = 0.777, *p* = 0.04) was associated with high ratings for spasm severity. When the TA EMG intensity was greater than that in SL (flexor peak for the SL/TA EMG ratio; ρ = −0.777, *p* = 0.04), and the RF EMG intensity was greater than for BF (extensor peak for the RF/BF EMG ratio; ρ = 0.777, *p* = 0.04), self-reported severity was greater. Both decreased BF EMG duration (ρ = −0.844, *p* = 0.017), and decreased coactivity (time) between RF and BF (ρ = −0.905, *p* = 0.005), were also associated with increased severity.

##### Pain and function

No significant correlations were found between transfer EMG and whether or not spasms were painful or interfered with function.

##### Tone

Intense EMG in SL (mean: ρ = 0.832, *p* = 0.023), TA (mean: ρ = 0.767, *p* = 0.044; peak: ρ = 0.954, *p* = 0.001), and BF (mean: ρ = 0.767, *p* = 0.044) was associated with high ratings for tone (Figure 7D). A high peak EMG intensity in TA in comparison to SL (flexor peak ratio for SL/TA) was also associated with greater self-reported tone (ρ = −0.917, *p* = 0.004).

##### Total self-reported rating

High intensity EMG in TA (mean: ρ = 0.893, *p* = 0.007; peak: ρ = 0.786, *p* = 0.036), RF (mean: ρ = 0.857, *p* = 0.014), and BF (peak: ρ = 0.857, *p* = 0.014) during the transfer was associated with a high total self-rating score. A greater peak EMG intensity in TA in comparison to SL (flexor peak ratio for SL/TA; ρ = −0.857, *p* = 0.014) and a greater RF peak EMG intensity in comparison to BF (extensor peak ratio for RF/BF; ρ = 0.857, *p* = 0.014) were also associated with a high total self-reported rating (Figure 7E). Other associations were not significant.

#### Physiology and Self-Report

No significant relationships were found between any of the physiological measures (H/M ratio, low frequency H-reflex depression, F/M area ratio and persistence) and self-report.

## Discussion

Our aims to quantify and associate three different measures of spasticity have shown that the involuntary EMG elicited during a transfer from a wheelchair to a bed (a daily functional task) correlated to both physiological and self-reported measures of spasticity. Decreased spinal inhibition and higher motoneuron excitability, measured physiologically by low frequency H-reflex depression and F/M area ratios, respectively, were both associated with long duration contractions during a transfer. In turn, reduced agonist-antagonist coactivity at both the ankle and knee joints (marked by less flexor activity) was associated with frequent or severe spasm ratings. Differences in intensity for agonist-antagonist muscle pairs (particularly in TA vs. SL, and RF vs. BF) related to severe spasms. Intense EMG related report of high tone. The many significant associations between our measures of spasticity suggest that quantification of EMG during naturally evoked involuntary contractions is an important new strategy to understand how physiological and self-report measures of spasticity relate to each other. Further, these results strongly support the idea that individuals with SCI describe spasticity in terms of their spasms and tone.

### Spinal Excitability and Inhibition Both Contribute to the Duration of Involuntary Muscle Activity after SCI Which Impacts Self-Report of Spasm Frequency

It may be easy to trigger and sustain activity in muscles paralyzed by SCI given the high motoneuron excitability reported here (Figure 4C) as well as changes in body position that commonly triggers spasms during daily tasks (Little et al., 1989). High TA F/M area ratios were associated with long BF EMG duration, with a similar trend for TA (ρ = 0.655, *p* = 0.111). A contraction triggered in one muscle may also facilitate contractions in other muscles leading to the high degree of co-activity seen throughout the transfers. For example, bursts of EMG in TA preceded extensive coactivation of leg muscles as participant 3 lay down (Figure 2E). The sustained firing of multiple muscles may continue relatively unchecked due to less low-frequency depression of the H-reflex (Figure 4B), reductions in other kinds of inhibition (Crone et al., 1994; Lee and Heckman, 2000; Nielsen et al., 2007), and/or from reciprocal facilitation (Crone et al., 2003; Xia and Rymer, 2005). To our knowledge, this is the first study to establish how levels of spinal excitation and inhibition after SCI relate to the duration of involuntary contractions during a natural task. We suggest that the duration of this muscle activity and/or the duration of agonist-antagonist coactivity may be used to count spasms. Near continuous SL EMG during transfers meant that activation of TA largely determined the time over which SL and TA were coactive. Both short duration TA EMG, and the consequent reduction in the duration of SL and TA coactivity, possibly from the lower intrinsic excitability of TA vs. SL motoneurons (Espiritu et al., 2003), were both associated with higher self-reported spasm frequency (Figure 7C). The duration of coactivity between muscles that act on the knee (RF and BF) also correlated to self-report of spasm frequency while severe spasms were reported when BF EMG duration was short. Thus, the timing of involuntary EMG elicited during a functional task, particularly in ankle and knee flexors, may drive how injured individuals describe the frequency and severity of their leg spasms (Figure 6B).

### Strong Contractions during Transfers Correlated with Severe Spasms, Tone and Overall Self-Report Scores

Strong associations between intense EMG in TA, RF and BF (but not SL) and severe spasms may arise from contractions that were as strong, or stronger than the voluntary contractions of uninjured subjects in a typical day. Notably, 86% of VL activity occurred at 5% MVC or less (Klein et al., 2010). Not only did average EMG intensities during transfers often reach 5% MVC (range for SL: 3.8–12.5% MVC, TA: 0.3–3.5%; RF: 0.6–6.6%; BF: 1.3–3.7%), peak EMG intensities were often several times greater. Intense contractions of TA and RF when unopposed by antagonists were also associated with high spasm severity and total self-reported spasticity scores, possibly because they resulted in ankle dorsiflexion and knee extension, respectively (Figure 3D). Provided sufficient force is generated to overcome inertia and gravity, joint movement and a perceivable spasm are more probable when a strong contraction (agonist) is met by weak or no opposition from an antagonist. For example, EMG intensity peaks for participant 3 reached 11% and 18% of uninjured MVC data in TA and RF, respectively, when SL and BF contractions were weak or non-existent. These intensities were within or exceeded the range of EMG intensities reported for over ground walking in uninjured people (Ericson et al., 1986; Ciccotti et al., 1994; Thomas et al., 2010). Consequently, this may be why this participant reported the most severe spasms. Further, this imbalance in EMG intensities occurred twice in TA and four times in RF during one transfer, which may produce multiple limb movements and increase perceived severity. Thus, the intensity of the involuntary EMG generated during daily activities, like transfers, may dictate how individuals with SCI perceive spasm severity and overall spasticity. This interpretation of correlations between the intensity of EMG in agonist vs. antagonist muscles, joint movements, and spasm severity ratings should be confirmed in future studies as joint angles and movement were not quantified here.

Intense EMG in all muscles (except RF) during transfers was associated with self-report of high tone. Experimenter-rated tone was also higher when EMG of higher amplitude was provoked by the passive joint movements used to evaluate tone during modified Ashworth tests (Sköld et al., 1998; Sherwood et al., 2000; McKay et al., 2004). Muscle shortening, increased stiffness, and/or changes in calcium metabolism after SCI (Howell et al., 1997; Mirbagheri et al., 2001; Lieber et al., 2004) increase motor unit twitch force after SCI (Griffin et al., 2002; Häger-Ross et al., 2006), so if submaximal muscle activation results in stronger contractions, participants may perceive them as increased tone. Strong activation of TA with little or no opposition from SL also explained a great amount of the variation in self-reported tone. When seated in a wheelchair, further dorsiflexion may stabilize the joint rather than cause movement, as may more balanced co-contraction of agonist and antagonist muscles which was common in our study. SCI individuals may interpret both of these situations as greater tone.

### Intense EMG during Transfers and Severe Spasm Ratings Occurred with Hyporeflexia

Quantification of physiological measures of spasticity unexpectedly revealed hyporeflexia in two of our SCI participants (Figure 4A) due to a disproportionate decrease in the H-reflexes vs. the M-waves. This hyporeflexia emphasizes that inputs from multiple afferents, not only Ia afferents, must drive spasms. Despite hyporeflexia in SL, participant 3 had remarkably high EMG intensity and coactivity during the transfer (Figures 2B,E) and also reported frequent and severe spasms. Participant 2 reported spasms of moderate severity with SL hyporeflexia (Figure 6A). Further, spasticity would decline with age if it were solely driven by Ia afferents, which is contrary to the literature (Sköld et al., 1999).

Only one SCI participant took baclofen (Table 1) so this medication cannot explain low H-reflex amplitudes. SCI itself does reduce Ia afferent input to motoneurons in injured rats (Soderblom et al., 2015) which could lower H-reflex amplitudes. However, our data from six SCI participants who had been injured for 1 or 2 years showed their SL H/M ratios (0.33–0.85) were similar to those of uninjured participants (0.41–0.84) in the same age range (19–38 years). The decrease in the H-reflex amplitude may therefore occur with age itself (Figure 5) or over time post SCI, possibly from retraction of new synapses formed in response to SCI, and/or differences in the type and amount of muscle use (deVries et al., 1985; Nielsen et al., 1993a; Lundbye-Jensen and Nielsen, 2008).

### More Excitable Lumbar Motoneurons after SCI Despite Varied EMG Duration and Intensity during Transfers

Supramaximal peripheral nerve stimulation antidromically activates a motoneuron to produce a F-wave provided there is sufficient depolarization for the impulses to travel into the soma-dendritic regions, and if the initial segment of the axon is not refractory (Eccles, 1955). Our F-wave area increase was greater than the decrease in M-wave area, relative to Uninjured data. Therefore, the significant increase in median F/M area ratios after SCI (Figure 4C) most likely resulted from more motoneurons responding to antidromic stimulation. A more depolarized resting membrane potential may generate F-waves in more motoneurons (Eccles, 1955; Beaumont et al., 2004). Prolongation of excitatory post-synaptic potentials after SCI (Norton et al., 2008), increases in the occurrence or amplitude of persistent inward currents in motoneurons (Bennett et al., 2001; Button et al., 2008), constitutive activation of 5-HT_2C_ receptors on motoneurons (Murray et al., 2010), and declines in multiple types of inhibition (e.g., Thomas and Field-Fote, 2009; Boulenguez et al., 2010) may also make motoneurons more responsive to peripheral nerve stimulation, resulting in higher F/M area ratios. Further, there may be preferential generation of F-waves in large motoneurons which typically produce stronger forces and larger EMG potentials (Zengel et al., 1985) because F-waves can be extinguished in slow conducting axons by reflex activity (Espiritu et al., 2003). SCI-induced motoneuron death is another possible contributor. Participant 5 had unusually low amplitude M-waves in TA (3.5% uninjured Mmax) which may reflect low motoneuron survival. F-waves in a small fraction of these units may elevate the F/M area ratio, but without much functional impact because participant 5 only had short, low intensity EMG during the transfer (Figure 2A) and reported no spasms (Figure 6A).

### Physiological Measures Vary Widely with or without SCI

Surprisingly, only high F/M area ratios distinguished individuals with SCI from uninjured participants (Figure 4C). No differences in the median SL H/M ratio and low frequency H-reflex depression were found because the distributions of data were wide and overlapped for both the SCI and uninjured groups (Figures 4A,B). Our participants with low H-reflexes also had greater low frequency H-reflex depression. If few motor units contribute to these low amplitude H-reflexes, inhibition of a small number of motoneurons may produce relatively more depression than reported previously (e.g., Calancie et al., 1993; Schindler-Ivens and Shields, 2000). Differences in inclusion criteria may explain the lack of change in SL H/M ratios with SCI. H/M ratios did not differ to uninjured data here, or in studies involving individuals with a similar level and duration of SCI, demographics (age, gender), and inclusion irrespective of the degree of spasticity (e.g., Ashby et al., 1974; Schindler-Ivens and Shields, 2004). Studies that have largely evaluated only SCI participants with severe spasticity have shown increased H/M ratios (Taylor et al., 1984; Calancie et al., 1993; Nielsen et al., 1993b).

### Individual Interpretation of Spasticity did Not Correlate with Physiological Indices of Spasticity

Motoneuron excitability can be high and/or inhibition can be reduced, but without a stimulus to activate the motoneurons, these physiological changes may not be apparent to the individual, whereas involuntary muscle contractions or changes in tone may be seen or felt. Even though only one of our participants had spared sensation in their legs and/or feet (the other three sensory incomplete participants only had sensory sparing at S3–S5; Table 1), and sensory scores from the AIS exam only indicate perceived light touch and pin prick, participants may well use sensory signals generated below the lesion that spread to the torso, for example, to register spasms and tone. Here, physiological indices were limited to two muscles, one type of inhibition, and largely focused on 1a afferents. Physiological measures in other leg muscles may increase and strengthen the correlations between physiological and functional measures of spasticity. However, even if involuntary EMG is used as an intermediary, relationships between physiology and self-report may remain undetected because no common features are measured. This may explain why significant associations between physiological and self-report measures have not been reported here or previously. Since our self-report data matched published data (83% of our participants experienced muscle spasms, 75% reported increased muscle tone, high spasm frequency strongly associated with severe spasms; Figure 6; Kawamura et al., 1989; Little et al., 1989; Lechner et al., 2006; Baunsgaard et al., 2016), and many people with SCI describe their muscle spasms when asked about spasticity (Mahoney et al., 2007), our EMG results reinforce that individuals with SCI likely use spasms and/or tone to rate their spasticity.

### Sample Size for Functional Measures of Spasticity and Correlations

Transfers are an important daily task for those who are wheelchair bound due to SCI because they are completed an average of 14 times a day (Finley et al., 2005). Although arm strength plays a large role in the ability to complete a transfer (Allison et al., 1995; Gagnon et al., 2005), maneuvering the trunk and legs onto a bed is integral to successful performance of this task, and 51% of individuals with SCI indicate it is made more difficult by spasticity (Little et al., 1989). Our sample size was reduced for the functional measures of spasticity since not all participants were able to transfer independently to our bed. However many strong and significant correlations were found between the functional, physiological, and self-reported measures of spasticity, even though the latter uses categorical data. Statistical power remained above 80% for all of the correlations despite the limited sample. These results indicate that the EMG evoked during a transfer in the laboratory may be a reasonable representation of the spasms that people with SCI notice because movement and changes in position (transfer) commonly triggers spasms (Figure 2), transfers are a common daily activity (Finley et al., 2005), and the evoked contractions can be prolonged and strong (Figure 3). The correlations between measures may even increase in strength if the resolution of the self-report scales were increased (severity, pain and interference with function were three point scales; frequency and tone were five point scales).

### Functional and Clinical Implications

Our results highlight that spasm counting may be a reasonable and relevant clinical measure of spasticity. First spasm frequency is indicative of spasm severity (Figure 6B). Second, high spasm frequency and painful spasms alone were associated with interference with function. Third, this correlation increased when the spasm frequency and pain scores were combined. It is also the individual with SCI who gauges the impact of spasticity on daily life, therefore assessments and treatments must start to be aimed at what matters most to the consumer (Mahoney et al., 2007). For example, complaint of frequent spasms or high tone, may indicate a need for interventions to reduce spasm duration or intensity, respectively. Interpretation of self-reported scores must also acknowledge that up to half of individuals with SCI find spasticity to be advantageous (Lechner et al., 2006), and many people develop strategies to cope with it (Yang et al., 1991; Voerman et al., 2009). If we are to capture a more comprehensive understanding of the complexities of spasticity, its effects on function, and targets for treatment, self-report measures must be coupled with functional analyses and measurement of the underlying biology.

## Conclusion

The results of this study provide evidence that relates physiological indices to individual perception of spasticity via the recording of involuntary EMG during a common daily activity (transfer from a wheelchair to a bed). Increased motoneuron excitability and decreased inhibition after SCI create an environment where diverse inputs may trigger long, intense involuntary muscle contractions. The timing and intensity of this activity is a large contributor to an individual’s perception of spasticity, especially if contractions are strong, and if there is an imbalance in both the duration and intensity of activity in agonist/antagonist muscles pairs (i.e., possible joint movement). Due to high variability, few differences in physiological indices were evident between people with and without SCI, therefore, focus must shift away from examining means to explaining variability, as well as hyporeflexia after SCI. The high degree of variability and strong correlations within our data further emphasize that multiple tests are necessary to fully capture both the biological basis and functional impact of spasticity.

## Author Contributions

Conception and design of experiments: MM, MC, CKT; Data acquisition, analysis and interpretation, manuscript writing, final approval of manuscript and agreement to be accountable for all aspects of the work: MM, BAD, MC, CKT.

## Funding

This research was funded by the Robert J. Kleberg, Jr. and Helen C. Kleberg Foundation, The Miami Project to Cure Paralysis, and a summer fellowship (to MM) from the National Institutes of Health (NS-083064).

## Conflict of Interest Statement

The authors declare that the research was conducted in the absence of any commercial or financial relationships that could be construed as a potential conflict of interest.
